# Molecular Dynamics Simulation of Hydrogen Permeation Behavior in Epoxy Resin Systems

**DOI:** 10.3390/polym17131755

**Published:** 2025-06-25

**Authors:** Chang Gao, Hongzhi Chen, Hao Xu, Zhanjun Wu, Xufeng Dong

**Affiliations:** 1 School of Materials Science and Engineering, Dalian University of Technology, Dalian 116024, China; rainy.gao723@mail.dlut.edu.cn (C.G.); dongxf@dlut.edu.cn (X.D.); 2 School of Aeronautics and Astronautics, Faculty of Vehicle Engineering and Mechanics, Dalian University of Technology, Dalian 116024, China; scottzz@mail.dlut.edu.cn

**Keywords:** liquid hydrogen storage, hydrogen permeation, composite tanks, molecular dynamics

## Abstract

Liquid hydrogen (LH_2_) storage using carbon-fiber-reinforced composite pressure vessels is facing increasing demands in aerospace engineering. However, hydrogen permeation in epoxy resin matrixes seriously jeopardizes the function and safety of the cryogenic vessels, and the micro-behavior of hydrogen permeation in epoxy resins remains mysterious. This study performed molecular dynamics (MD) simulations to investigate the hydrogen molecule permeation behaviors in two types of epoxy resin systems, with similar epoxy reins of bisphenol A diglycidyl ether (DGEBA) and different curing agents, i.e., 4,4′-diaminodiphenylmethane (DDM) and polypropylene glycol bis(2-aminopropyl ether) (PEA). The influencing factors, including the cross-linking degrees and temperatures, on hydrogen permeation were analyzed. It was revealed that increased cross-linking degrees enhance the tortuosity of hydrogen diffusion pathways, thereby inhibiting permeation. The adsorption characteristics demonstrated high sensitivity to temperature variations, leading to intensified hydrogen permeation at low temperatures. By triggering defects in the epoxy resin systems by uniaxial tensile simulation, high consistency between the simulation results and the results from helium permeability experiments can be achieved due to the micro-defects in the simulation model that are more realistic in practical materials. The findings provide theoretical insights into micro-scale permeation behavior and facilitate the development of high-performance epoxy resins in liquid hydrogen storage.

## 1. Introduction

Hydrogen storage technology has witnessed a significant evolution from all-metal to carbon-fiber-reinforced composite pressure vessels, aiming to meet the growing demands for efficient hydrogen storage in various applications, especially in the aerospace and automotive industries [1,2]. Currently, Type I and Type V vessels are viable options for liquid hydrogen (LH_2_) storage. Type I vessels are typically constructed from metals like stainless steel, aluminum, or titanium, which have a substantial dead weight. Type V vessels, made entirely of carbon-fiber-reinforced polymer (CFRP), rely solely on the CFRP for structural integrity and gas-barrier properties [3,4]. The absence of a traditional liner exposes them to issues such as cryogenic mechanical failure and hydrogen leakage.

Carbon fibers exhibit outstanding chemical stability and physical properties [5], remaining largely unaffected by cryogenic and high-pressure working conditions. Consequently, the epoxy resin matrix becomes the critical determinant of the overall performance of LH_2_ vessels. The leakage of LH_2_ into the epoxy resin matrix can result in functional failure of the vessels. Moreover, repeated cryo-thermal cycles in their operating environment (with the temperature of LH_2_ being −253 °C) induce thermal stresses in the CFRP, leading to the formation and propagation of micro-cracks, which can eventually cause defects [6] that significantly reduce the remaining lives of the vessels, posing safety risks and limiting their practical application. Therefore, it is of great importance to enhance the cryogenic mechanical performance and impermeability of epoxy resin matrixes.

The conventional research and development (R&D) process of epoxy resin is highly reliant on experimental methods. Nevertheless, this approach frequently encounters two significant challenges: an extended R&D cycle and substantial economic costs. Epoxy resin features a complex cross-linking network, and numerous factors can influence its structure and properties. Furthermore, the intricate interplay and correlations among these influencing factors pose a formidable challenge for experimental methods, making it difficult to precisely elucidate the relationship between the macroscopic properties and microscopic mechanisms of epoxy resin [7,8,9].

By leveraging the power of computational algorithms, molecular dynamics (MD) can model the behavior of epoxy resin systems at the atomic and molecular levels with remarkable precision. This enables not only the visual analysis of microstructures—such as the cross-linking network in epoxy resins—but also unravels the fundamental mechanisms governing macroscopic properties like mechanical strength and low-temperature embrittlement through quantitative simulation calculations. By achieving this, it effectively overcomes the technical limitations of traditional experimental approaches in characterizing extreme operational conditions and observing micro-scale processes [10,11,12]. By leveraging algorithms developed for the generation of reasonable cross-linked networks, MD simulations have been carried out to predict a variety of thermomechanical properties of epoxy thermosets, including glass transition temperature (Tg), Young’s modulus, coefficient of thermal expansion (CTE), yield behavior, etc. By considering the effects of strain rate and temperature, the simulated results show satisfactory consistency with experimental data.

Many scholars have investigated the permeation behaviors of H_2_ in various materials through MD simulations. For example, Zheng et al. [13] employed MD simulations and Grand Canonical Monte Carlo (GCMC) methods to study the solubility and diffusion characteristics of hydrogen in amorphous polyethylene (PE) under pressures of 0.1–0.7 MPa at room temperature. They found that the solubility, diffusion coefficient, and permeation coefficient of hydrogen in amorphous PE increase with rising temperature. Liu et al. [14] utilized MD simulations and MD-MC algorithms to analyze hydrogen leakage through shale caprocks, examining the effects of temperature, pore size, and pressure on leakage behavior. Hassanzadeh et al. [15] calculated the solubility, diffusion coefficients, and permeability of pure gases and binary gas mixtures in MFI (Mobil-Five) zeolites, exploring how pressure and temperature influence transport properties. Their results showed that hydrogen’s solubility coefficient decreases with increasing temperature, while the diffusion coefficient exhibits the opposite trend.

Most existing hydrogen permeation simulations focus on room-temperature environments, with few reporting on hydrogen permeation in polymers at liquid hydrogen storage temperatures (20 K). This work combines MD simulations with GCMC methods to systematically analyze hydrogen permeation in epoxy resin systems under different cross-linking degrees and temperature conditions. Experimental validation is also applied to provide a robust theoretical framework and practical techniques for accurately predicting the hydrogen permeation performance of epoxy resins in complex environments [16,17,18,19].

This study conducted MD simulation of hydrogen molecule permeation behavior in DGEBA/DDM and DGEBA/PEA epoxy resin systems. The influence mechanisms of key factors, including the cross-linking degrees and temperatures, were systematically analyzed. The research revealed that increased cross-linking degrees enhance the tortuosity of hydrogen diffusion pathways, thereby inhibiting permeation. The adsorption characteristics demonstrated high sensitivity to temperature variations, leading to intensified hydrogen permeation at low temperatures. Finally, the permeation simulation results were compared with the results from a helium permeability experiment. It was found that by triggering damage evolution in the epoxy resin systems by uniaxial tensile simulation, high consistency between the simulation and experimental results can be achieved due to the micro-defects in the simulation model that are more realistic in practical materials. The findings provide theoretical insights into micro-scale permeation behavior and technical guidance to facilitate the engineering applications of epoxy resin materials in liquid hydrogen storage tanks.

## 2. Materials and Methods

### 2.1. Molecular Dynamics Simulation

#### 2.1.1. Model Preparation

**Figure 1:**
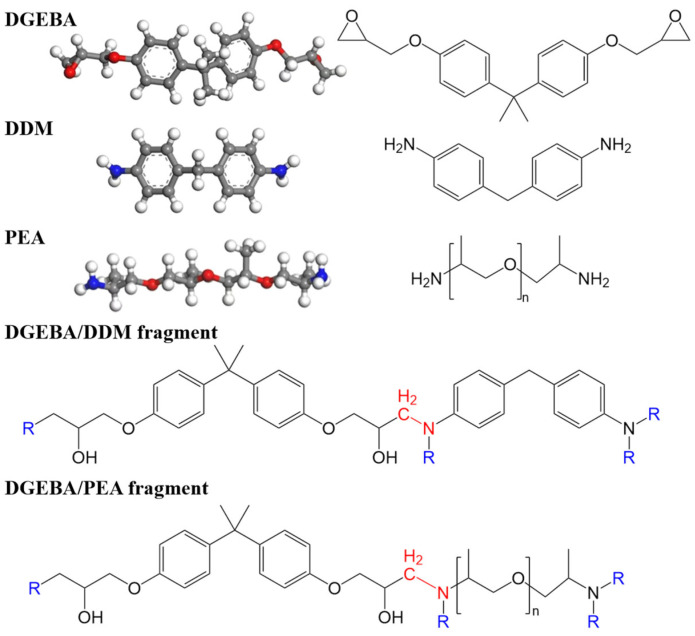
The molecular structures and models for DGEBA, DDM, PEA, and the cross-linking fragments.

**Figure 2:**
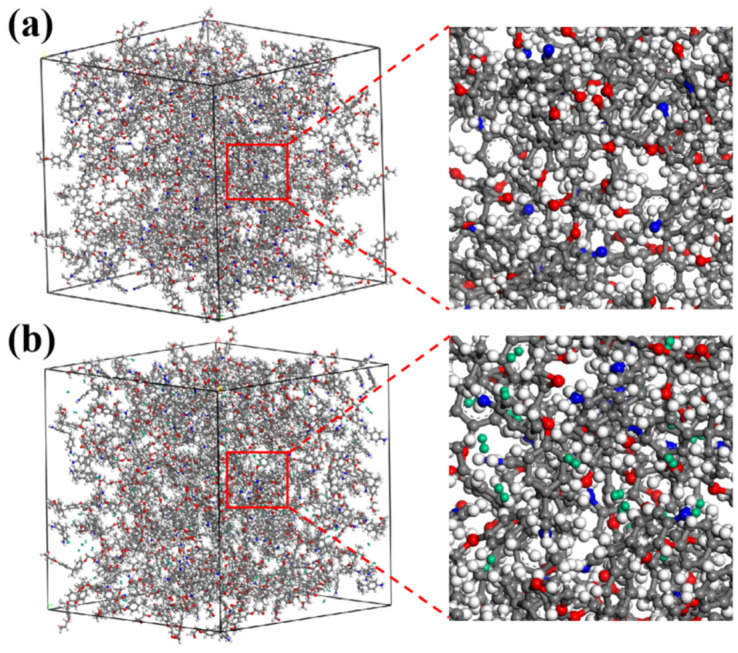
(**a**) Adsorption pool model; (**b**) diffusion pool model.

The models of hydrogen molecules, epoxy resin monomer diglycidyl ether of bisphenol A (DGEBA), and curing agents 4,4-diaminodiphenylmethane (DDM) and polypropylene glycol bis(2-aminopropyl ether) (PEA) were generated using Accelrys Materials Studio. The chemical structures are shown in Figure 1. To investigate the hydrogen permeation behavior of epoxy resins, adsorption and diffusion models were constructed by packing them in periodic boxes with desired compositions. Specifically, the adsorption pool model was composed of 480 DGEBA monomers and 240 curing agent monomers (DDM or PEA). The diffusion pool model added 480 hydrogen molecules on the basis of the adsorption pool. The green structures in Figure 2b represent hydrogen molecules.

MD simulations were carried out using the open-source package LAMMPS and the PCFF force field. The Polymatic algorithm developed by Abbott et al. [19] was applied to mimic the curing process using a distance-based criterion for chemical reactions. In detail, the curing agents and activated DGEBA epoxy resins were blended by packing them in periodic boxes. The distance between the eligible nitrogen in the -NH_2_ group of the DDM/PEA and the carbon atom in the activated epoxy group of DGEBA was calculated every few timesteps, and a covalent bond was formed between the atom pair when the inter-atomic distance was smaller than a cutoff distance of 6 Å. The temperature was set to 600 K to accelerate the reaction. Structure optimization and dynamic equilibration were applied frequently to guarantee low residual stresses in the system. The reaction process was terminated once the target cross-linking degree was reached, around 90% in the present study. The adsorption and diffusion models were cured under 600 K, followed by a programmed temperature-controlled annealing process (the annealing rate was 0.005 K/s). Under the NPT ensemble, with a temperature gradient of 10 K, the system was gradually cooled from 600 K to 20 K (at a constant pressure of 1 atm). A multi-level relaxation cycle of 200 ps was performed at each temperature (residual stress was eliminated through multi-step energy minimization and kinetic simulation to achieve volume balance), and a series of models covering a temperature range from 300 K to 20 K were ready for subsequent properties simulation.

#### 2.1.2. Simulation of Permeation

The permeation behavior of epoxy resins can be characterized by the permeability coefficient (P). According to the solution adsorption model, the permeability coefficient is dependent on its solubility and diffusion ability [20]. It can be derived by the following expression:

(1) P=S×D

where S is the solubility coefficient with the unit of ${cm}^{3}\;(STP)\cdot {cm}^{-3}\cdot {Pa}^{-1}$, and D is the diffusion coefficient with the unit of ${cm}^{2}\cdot s^{-1}$. The unit of P is ${cm}^{3}\;(STP)\cdot cm\cdot {cm}^{-2}\cdot s^{-1}\cdot {Pa}^{-1}$.

The solubility coefficient S is defined as the concentration at which medium molecules dissolve within an epoxy resin matrix upon achieving thermodynamic equilibrium under a unit of pressure. Fundamentally, this coefficient serves as a quantitative measure of the thermodynamic compatibility between the medium molecules and the resin system. It encapsulates the intricate interplay of intermolecular forces, such as van der Waals forces, hydrogen bonding interactions, and dipole–dipole attractions, that govern the extent to which the medium can disperse and stably exist within the epoxy resin structure. It can be measured by the adsorption isotherm method. It is defined as the limiting slope of the adsorption isotherm [21,22], i.e.:

(2) $$S=\underset{f\to 0}{\lim}\frac{C}{f}=K_{H}$$

where C represents the dissolved concentration of gas molecules in the polymer, with the unit of ${cm}^{3}\;(STP)\cdot {cm}^{-3}$; $K_{H}$ is Henry’s constant; and f stands for the fugacity with the unit of Pa. Among the three, $C=K_{H}f$.

The diffusion coefficient D is a key kinetic parameter that quantifies the migration rate of medium molecules within a polymer matrix. It can be determined by analyzing the mean squared displacement (MSD) of molecular motion. This calculation assumes that the diffusion phenomenon is limited to the amorphous part of the polymer and that the diffusion region remains consistent. In the MD simulation method, the relationship between the MSD and the duration of molecular motion is established by tracking the trajectory of the center of the permeating molecule [23]. The expression is as follows:

(3) $$D=\frac{1}{6N}\underset{t\to \infty }{lim}\frac{d}{dt}\sum_{i=1}^{N}{\left[r_{i}(t)-r_{i}(0)\right]}^{2}$$

where N is the number of molecules; $r_{i}\left(0\right)$ and $r_{i}(t)$ are the initial and final positions of the center of mass of the molecule at the time interval t, respectively; and ${\left[r_{i}(t)-r_{i}(0)\right]}^{2}$ is the average value of the MSD of the molecules. The MSD is calculated by averaging over N data points. According to the Einstein formula, Equation (3) can be simplified as:

(4) D=a/6

where a is the slope of the straight line obtained by fitting the MSD relationship curve using the least squares method [24].

#### 2.1.3. Simulation of Free Volume

Free volume is an important concept in the field of materials science for describing the microscopic structure of amorphous materials such as polymers and glasses. It refers to the movable gaps within the material that are not occupied by atoms or molecules. These gaps provide channels for the movement of molecular chain segments and the diffusion of small molecules and are core elements determining the dynamic behavior of materials such as diffusion, permeation, and mechanical relaxation.

The open-source Zeo++ software [25,26,27] for high-throughput analysis of porous materials can introduce a large number of spherical probes into the simulation system. By statistically analyzing samples of different sizes, it can determine the accessible occupied volume that the probes in the model can approach. These accessible occupied volumes (POAV) can be approximately regarded as various tiny voids in the system. Based on this, by calculating the volume fraction of POAV in the whole system, the distribution and evolution of the internal free volume of the two epoxy resin systems under different cross-linking degrees can be obtained.

### 2.2. Experimental Procedure

#### 2.2.1. Preparation of Epoxy Resins

A calculated amount of curing agent (DDM or PEA) was added to DGEBA epoxy resin under stirring at 80 °C until a homogeneous mixture was obtained. After degassing in a vacuum oven, the resulting mixture was then poured into a Teflon mold and cured at 100 °C for 2 h, 130 °C for 2 h, and 160 °C for 4 h.

#### 2.2.2. Helium Permeability Experiment

**Figure 3:**
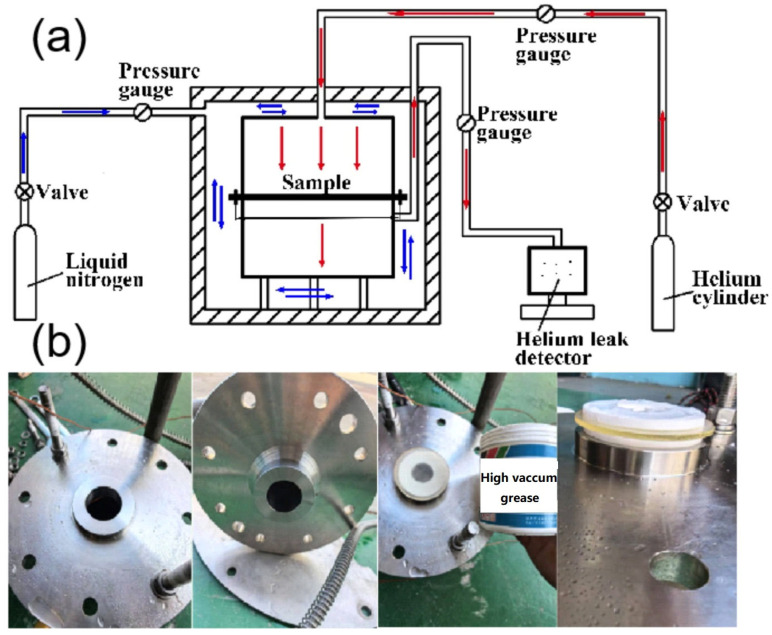
The schematic diagram (**a**) (blue arrows stand for the current of liquid nitrogen and red ones stand for the current of helium) and photograph (**b**) of the experimental setup for the helium permeation test.

Due to the highly flammable and explosive nature of hydrogen, conducting hydrogen permeation experiments poses significant safety risks. Therefore, this study employed an experimental approach that integrated pressurized helium with liquid nitrogen cooling to investigate the helium permeation behavior of epoxy resin systems under low-temperature conditions. The schematic diagram of the experimental setup and a photograph of the equipment are illustrated in Figure 3.

Helium was utilized as the pressurizing medium in the upper chamber of the experimental apparatus. The research obtained the relationship between helium leakage and temperature by measuring the helium leakage rates of the target samples at both room temperature and liquid nitrogen temperature. The entire experimental procedure was meticulously divided into four distinct phases: pre-experiment preparations, helium pressurization, data collection using a helium mass spectrometer detector, and post-experiment processing.

**Figure 4:**
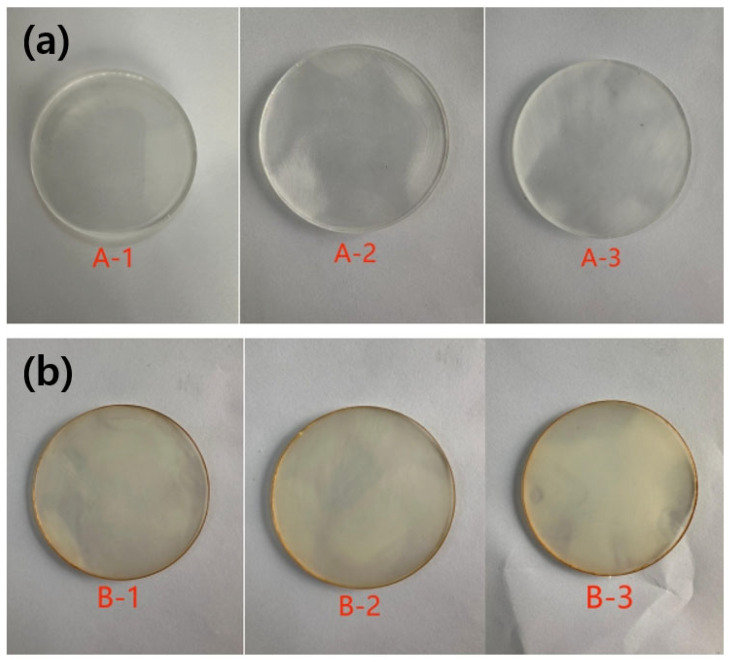
Helium permeation test specimens: (**a**) DGEBA/PEA system; (**b**) DGEBA/DDM system.

Two sets of epoxy resin samples (DGEBA/DDM and DGEBA/PEA) were prepared, which were circular discs with a diameter of 27.5 mm and a thickness of 2 mm (Figure 4). For each set, three parallel samples were prepared to ensure the reliability and reproducibility of subsequent experimental results.

During the test preparation process, the sealing surfaces of the upper and lower chambers of the experimental apparatus had to be meticulously wiped with anhydrous ethanol to ensure pristine cleanliness. Concurrently, high-vacuum silicone grease had to be uniformly applied to the interface between the sample and the sealing gasket. Subsequently, the sealing gasket and the sample were placed on the sealing surface of the lower chamber in a sequential manner.

Once the above procedures were completed, a thorough inspection of the equipment pipelines’ operational status was conducted. Only after confirming that there were no anomalies was the helium mass spectrometer detector activated, and both the upper and lower chambers were evacuated simultaneously. The shut-off valve of the pipeline in the helium mass spectrometry detection segment was then opened, and the pressure value of the absolute pressure gauge was monitored in real-time. When the pressure reading dropped below 100 Pa, the initial helium concentration data of the lower chamber was recorded while the upper chamber remained unfilled with helium.

The entire experimental setup was subsequently placed in a liquid nitrogen thermal insulation bucket, and the automatic temperature compensation function was engaged. This enabled rapid cooling via the automated injection of liquid nitrogen. Throughout the testing process, the temperature sensor data was continuously monitored. Upon the temperature reaching the target test value, the helium pressurization protocol was initiated, and the helium pressure within the upper chamber was increased gradually. Once the pressure stabilized, the helium detection data from the lower chamber was promptly read and recorded.

The leakage rate is a fundamental physical quantity that quantifies the rate at which gas leaks through materials or sealing structures. It is rigorously defined as the product of the gas volume flow rate passing through material flaws or interfacial defects per unit time and the applied pressure under specified differential pressure conditions, expressed in $Pa\cdot m^{3}/s$.

The Gas Transmission Rate (GTR), on the other hand, serves as a pivotal parameter for characterizing a material’s ability to allow gas permeation. Specifically, GTR is defined as the volume of gas that traverses a unit area and unit thickness of the material per unit time under standard conditions and a unit pressure differential, with its unit being ${cm}^{3}\;(STP)\cdot {cm}^{-2}\cdot d^{-1}\cdot {Pa}^{-1}$. This parameter offers a comprehensive assessment of a material’s gas barrier properties and stands as a cornerstone metric for evaluating the airtightness of sealing materials, such as epoxy resins.

In accordance with the stipulations of the national standard GB/T 1038.1-2022 [28], the gas permeation amount of an epoxy resin system can be systematically converted into the GTR using Equation (5). Subsequently, through Equation (6), it can be further transformed into the gas permeation coefficient.

(5) $$GTR=\frac{\Delta p}{\Delta t}\times \frac{V}{A}\times \frac{T_{0}}{p_{0}T}\times \frac{24}{p_{1}-p_{2}}=\frac{L\cdot 8.64\times 10^{10}}{A\cdot \Delta p}$$

(6) $$P=\frac{\Delta p}{\Delta t}\times \frac{V}{A}\times \frac{T_{0}}{p_{0}T}\times \frac{d}{p_{1}-p_{2}}=1.1574\times 10^{-7}GTR\times d$$

where Δp/Δt represents the arithmetic mean of the pressure variation in the low-pressure chamber per unit time during the steady-state gas permeation process, measured in Pa/h; V denotes the volume of the low-pressure chamber (unit: ${cm}^{3}$); A is the area of the sample (unit: $m^{2}$); $T_{0}$ and $p_{0}$ signify the temperature (273.15 K) and pressure ($1.0133\times 10^{5}\;Pa$) under standard conditions, respectively; T indicates the test temperature (unit: K); $p_{1}-p_{2}$ represents the pressure difference across the sample (unit: Pa); and d stands for the thickness of the sample (unit: m).

## 3. Results and Discussion

### 3.1. Effect of Cross-Linking Degree on Hydrogen Permeation in Epoxy Resins

#### 3.1.1. Solubility Coefficient

To study the effect of the cross-linking degree on the hydrogen permeation behavior in epoxy resins, adsorption pool models of DGEBA/DDM and DGEBA/PEA epoxy resins with a gradient cross-linking degree (ranging from 70% to 90% and incrementing at intervals of 5%) were constructed, respectively. The adsorption isotherms of liquid hydrogen in various resin systems were quantitatively calculated using the Sorption module in Materials Studio based on the Grand Canonical Monte Carlo Method (GCMC) [29].

**Figure 5:**
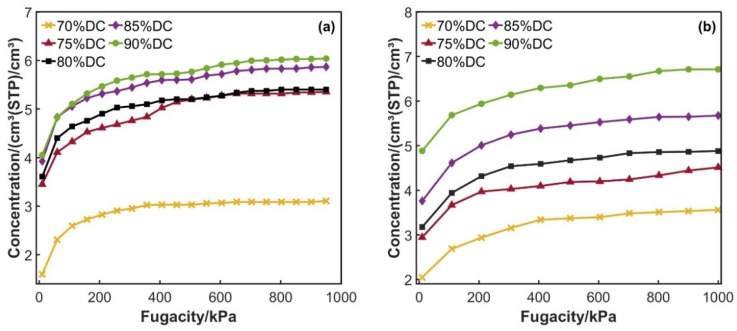
Sorption isotherm of $H_{2}$: (**a**) DGEBA/PEA system; (**b**) DGEBA/DDM system.

As depicted in Figure 5, there is approximately a significant linear relationship between the adsorption concentration of liquid hydrogen and the fugacity at 20 K (liquid hydrogen storage temperature). The solubility coefficient of liquid hydrogen can be characterized as the limiting slope of the adsorption isotherm under equilibrium conditions. The specific values of the solubility coefficients for the DGEBA/PEA and DGEBA/DDM epoxy resin systems are presented in Table 1 and Table 2.

It can be seen that in both epoxy resin systems, the solubility coefficient shows an upward trend with the increase in the cross-linking degree. This phenomenon might seem to conflict with the traditional free volume theory. However, in reality, it unveils the regulatory mechanism of the microscopic chemical environment of the material on the dissolution behavior [30,31]. Within the liquid hydrogen storage temperature range (20 K), the segment motion of the epoxy resin system is frozen, and the dynamic redistribution mechanism under the free volume is notably restricted by the temperature. The curing agents contain amino groups (-NH_2_). When the cross-linking degree increases, the density of amino groups per unit volume increases, and an interaction occurs between the lone pair electrons of the amino groups and the H atoms of hydrogen molecules (H_2_), forming a weak interaction similar to a hydrogen bond [32]. The low-temperature and high-pressure conditions not only decelerate the thermal motion of the molecules but also boost the concentration of hydrogen molecules. This further promotes the formation and stabilization of this weak interaction, thereby enhancing the adsorption and solubility of hydrogen molecules within the system.

The variation in the cross-linking degree has a profound impact on the three-dimensional network structure and mechanical properties of the system. In the DGEBA/PEA system, the change range of the solubility coefficient with the increase in the cross-linking degree is 34.21%, whereas in the DGEBA/DDM system, it is merely 28.33%. This suggests that the adsorption behavior of hydrogen molecules is relatively less sensitive to the cross-linking degree. Its dominant mechanism is likely the non-specific adsorption of hydrogen molecules by the polar groups in the epoxy resin, which is closely related to both the inherent characteristics of the medium itself and the external environmental factors.

#### 3.1.2. Diffusion Coefficient

**Figure 6:**
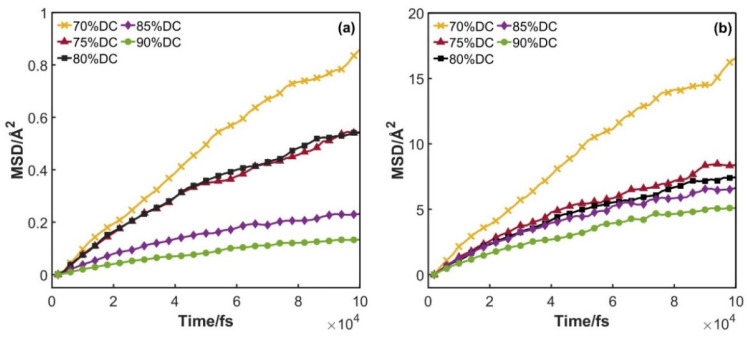
Variation in MSD with time: (**a**) DGEBA/PEA system; (**b**) DGEBA/DDM system.

Diffusion pool models of epoxy resins (DGEBA/DDM and DGEBA/PEA) with a gradient in the cross-linking degree (increasing from 70% to 90% in 5% increments) were constructed. Figure 6 illustrates the curves depicting how the mean squared displacement (MSD) of hydrogen molecules in the two epoxy resin systems varies with time across different cross-linking degrees. The diffusion coefficient of hydrogen molecules can be defined as the slope of the straight line obtained by fitting the MSD parameter in the epoxy resin system using the least squares method. Thus, the diffusion coefficients of hydrogen molecules in the DGEBA/DDM and DGEBA/PEA epoxy resin systems at various cross-linking degrees were obtained, and the specific values are presented in Table 1 and Table 2.

It can be observed that in both epoxy resin systems, when the cross-linking degree increases from 70% to 90%, the diffusion coefficient shows a significant downward trend. According to the “tortuosity factor” mechanism in the permeation theory [33,34], as the density of cross-linking points increases, the three-dimensional network obstacles formed make the diffusion paths inside the resin more tortuous. Specifically, when hydrogen molecules diffuse, they need to bypass these cross-linking points multiple times, which leads to an increase in the effective migration path and thus a decrease in the diffusion rate.

A more thorough analysis indicates that an increase in the cross-linking degree enhances the interaction between polymer chains, thereby restricting the local motion (such as vibration or rotation) of chain segments. This diminished mobility reduces the material’s capability to reconfigure its dynamic microvoids, effectively creating a more stable diffusion barrier. Notably, as stated in references [35,36], a higher cross-linking degree typically elevates the glass transition temperature (Tg) of the epoxy resin. This causes the material to approach a glassy state more closely at the same temperature. In this glassy state, the movement of chain segments is severely restricted, and the free-volume distribution becomes more rigid, ultimately leading to a substantial decrease in the diffusion coefficient. Although epoxy resin is a thermosetting material, the topological changes induced by cross-linking compact the amorphous region’s structure, reducing molecular spacing and impeding the permeation of hydrogen molecules.

#### 3.1.3. Permeability Coefficient

According to Equation (1), the permeability coefficients of hydrogen in the PEA and DDM epoxy resin systems can be calculated, and the specific values are shown in Table 1 and Table 2. It can be seen that when the cross-linking degree of the two epoxy resin systems increases from 70% to 90%, the permeation rate of hydrogen molecules in the material shows a significant upward trend, which is highly consistent with the change in the diffusion coefficient with the cross-linking degree. This fully indicates that in the epoxy resin system regulated by the cross-linking degree, the permeation behavior of hydrogen molecules is mainly dominated by the diffusion process.

Combined with the previous analysis of the adsorption characteristics, the adsorption effect of the epoxy resin system on hydrogen has a low sensitivity to the change in the cross-linking degree. Specifically, the results of molecular dynamics simulations show that the solubility coefficients of systems with different cross-linking degrees only fluctuate within a small range. The change mechanism may be related to the transformation of the distribution state of the free volume of the system from a dynamic state to a static state, and this change in the topological structure can largely restrict the permeation behavior of liquid hydrogen molecules.

#### 3.1.4. Free Volume

**Figure 7:**
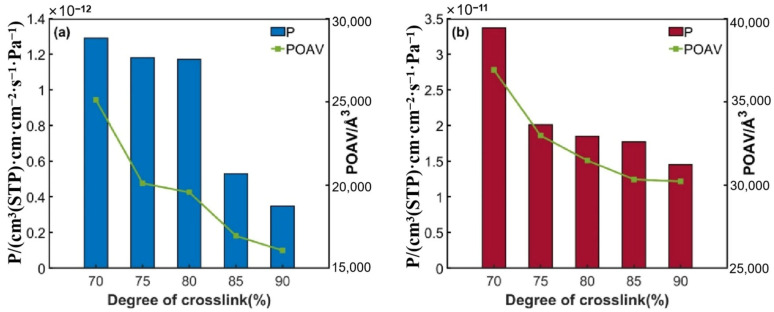
Variation in permeation coefficient and POAV with cross-linking degree: (**a**) DGEBA/PEA system; (**b**) DGEBA/DDM system.

As can be seen from Figure 7, as the cross-linking degree increases from 70% to 90%, the POAV inside the two epoxy resin systems shows a downward trend, and the free volume decreases accordingly. This fully demonstrates that the evolution of the free volume will affect the barrier performance of the material medium. The increase in the cross-linking degree promotes the molecular chains in the system to form a more compact three-dimensional network structure, thus reducing the internal free volume of the material. The smaller the free volume, the smaller the permeability coefficient of small molecular media such as liquid hydrogen, and the more difficult it is for the permeation behavior to occur.

In addition, this phenomenon also reveals the existence of dual spatial distribution characteristics within epoxy resins. On one hand, there are dense regions formed by the close packing of molecular chains. On the other hand, there are micro-scale free volume pores discretely distributed within the material. These tiny voids merge with each other to form permeation channels for the movement of medium molecules. Experimental data show that the diffusion behavior of small molecules of low-temperature media such as liquid hydrogen is mainly restricted by the topological structure of the free volume in the three-dimensional cross-linked network and its dynamic evolution characteristics.

It is worth noting that when the cross-linking degree is increased to the critical threshold, the connectivity between the free volumes may change abruptly. The movement between the chain segments is restricted, which, in turn, hinders the reconstruction ability of the dynamic microvoids within the system, resulting in a rapid decline in the permeability coefficient. This provides a theoretical basis for predicting and optimizing the liquid hydrogen barrier performance of epoxy resins at the macroscopic level by regulating the microscopic network structure of epoxy resins, such as the free volume distribution and the density of cross-linking points.

### 3.2. Parametric Discussion

#### 3.2.1. Effect of Temperature on Hydrogen Permeation

**Figure 8:**
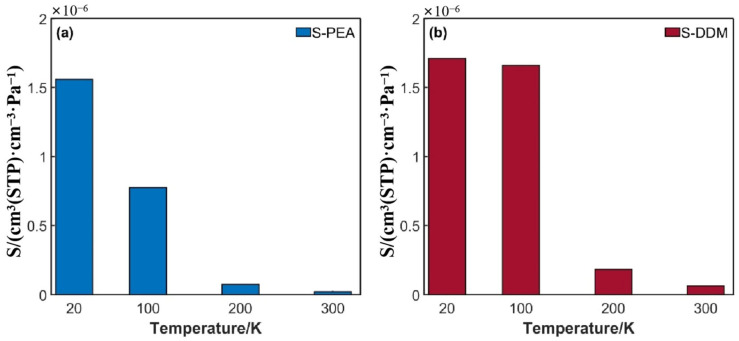
Variation in solubility coefficient with temperature: (**a**) DGEBA/PEA system; (**b**) DGEBA/DDM system.

DGEBA/PEA and DGEBA/DDM epoxy resin systems with a cross-linking degree of 90% were constructed. These models incorporated both an adsorption model and a diffusion model. Based on these models, a systematic investigation was conducted to explore the influence of temperature on the hydrogen barrier properties of the materials. By comparing and analyzing the evolution curves of the hydrogen molecule solubility coefficients (Figure 8) and diffusion coefficients (Figure 8) of the two sets of epoxy systems at four characteristic temperatures, i.e., 20 K, 100 K, 200 K, and 300 K, the microscopic mechanisms of hydrogen molecule adsorption kinetics and diffusion migration behavior within the epoxy resins under temperature gradients were revealed.

As depicted in Figure 8, the adsorption behavior of hydrogen molecules exhibits a remarkable temperature dependence, with the solubility coefficient showing a distinct decreasing trend as the temperature rises. According to Le Chatelier’s principle [37], an increase in temperature drives the equilibrium towards the endothermic direction (i.e., the escape of gas from the system), thereby leading to a decrease in solubility. As described in Section 3.1.1, at lower temperatures, the amino functional groups in both the DGEBA/PEA and DGEBA/DDM epoxy resin systems form a weak interaction similar to a hydrogen bond with hydrogen molecules. When hydrogen molecules move within the system, this weak interaction makes them more easily captured. Conversely, an increase in temperature further weakens the interaction between the amino functional groups and hydrogen molecules.

From the perspectives of molecular dynamics and thermodynamics, the average kinetic energy of hydrogen molecules increases with rising temperature. Adsorption sites have a certain potential well depth. When the kinetic energy of hydrogen molecules exceeds this potential well depth, hydrogen molecules are more likely to desorb from the adsorption sites and subsequently escape from the epoxy resin system. Moreover, as the temperature continues to rise, the intermolecular forces within polymer systems weaken. Although the cross-linked network restricts the movement of macromolecular chain segments to some extent, it still intensifies the vibration and rotation of local chain segments. This increases the dynamic disorder of adsorption sites, making it difficult for hydrogen molecules to stably remain at the adsorption sites, thus shortening their effective residence time.

**Figure 9:**
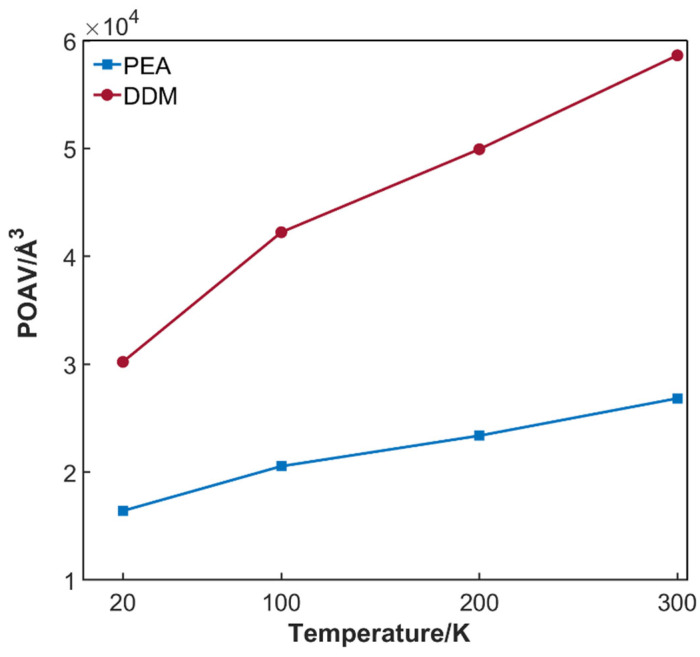
POAV trend of DGEBA/PEA and DGEBA/DDM systems at different temperatures.

Analyzing from the perspective of changes in internal free volume of the system, as shown in Figure 9, with the increase in temperature, the internal free volume of the epoxy resin system expands due to the dynamic changes in chain segments. The increase in free volume allows hydrogen molecules to move more freely within the epoxy resin system, reducing the direct contact opportunities between hydrogen molecules and the system, thereby decreasing the system’s ability to adsorb hydrogen molecules. It is particularly important to note that when the temperature approaches the glass transition temperature of the epoxy resin, the adsorption coefficient of the epoxy resin for hydrogen molecules decays exponentially. Specifically, when the epoxy resin transitions from the glassy state to the rubbery state, the movement pattern of molecular chains changes significantly. In the glassy state, the movement of molecular chains is highly restricted, mainly manifested as local oscillations. In contrast, in the rubbery state, molecular chains gain greater freedom and can undergo more extensive coordinated movements. These internal changes in the system create a competitive mechanism with hydrogen molecule adsorption, thereby disrupting the stable adsorption relationship between hydrogen molecules and the system.

**Figure 10:**
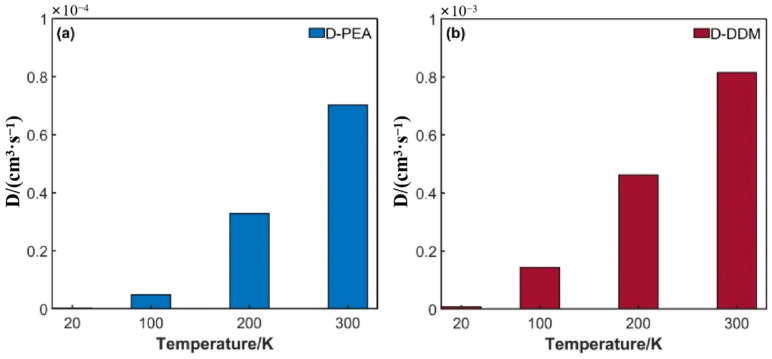
Variation in diffusion coefficient with temperature: (**a**) DGEBA/PEA system; (**b**) DGEBA/DDM system.

As shown in Figure 10, the diffusion behavior of hydrogen molecules in the epoxy resin system exhibits a significant temperature dependence, and its diffusion coefficient shows an exponential growth trend with the increase in temperature. This temperature sensitivity stems from the synergistic effect of multiple mechanisms: temperature serves as a macroscopic measure of the kinetic energy of molecular thermal motion. When the temperature of the system decreases, the kinetic energy of hydrogen molecules decays significantly, resulting in a decrease in the frequency of their diffusion and migration; at the same time, the thermodynamic energy of the system is insufficient to overcome the activation energy barrier of the coordinated motion of molecular chain segments, and the motion of polymer chain segments is restricted. Since the free volume is composed of transient voids generated by the motion of molecular chain segments, the restricted motion of chain segments directly leads to the shrinkage of the free volume size and a decrease in its density. At this time, the free volume distribution enters a “frozen state” and loses its dynamic adjustment ability, significantly increasing the tortuosity of the diffusion path of hydrogen molecules. The coupled effects of the above-mentioned thermodynamics and kinetics jointly lead to the obstruction of the diffusion channels of hydrogen molecules and a reduction in the diffusion efficiency.

Conversely, as the temperature rises, the input of thermal energy enhances the thermal activation effect of the molecular chains. The molecular chains obtain enough activation energy to overcome the rotational barrier, and their coordinated motion is significantly enhanced, promoting the dynamic reconstruction of free volume voids; meanwhile, the motion amplitude of chain segments increases, expanding the effective size of the diffusion channels and forming a continuous diffusion network. This thermodynamically driven evolution of the microstructure significantly improves the diffusion efficiency of hydrogen molecules. When the temperature approaches the critical point of the glass transition, the epoxy resin transforms from the glassy state to the rubbery state, the degree of freedom of molecular chains increases sharply, and the coefficient of coordinated thermal expansion changes significantly, further accelerating the growth rate of the free volume. This dual effect leads to a sudden expansion of the diffusion channels, ultimately causing a leap in the diffusion coefficient.

**Figure 11:**
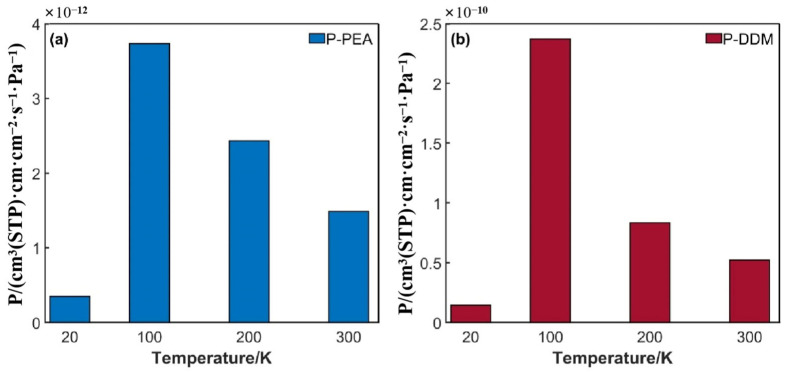
Variation in permeability coefficients with temperature: (**a**) DGEBA/PEA system; (**b**) DGEBA/DDM system.

Figure 11 shows the variation in the hydrogen permeation coefficient with temperature in the DGEBA/PEA and DGEBA/DDM epoxy systems. The results indicate that when the temperature drops from 300 K to 100 K, the hydrogen molecule permeation coefficient exhibits a significant upward trend, which highly coincides with the changes in the adsorption behavior of hydrogen molecules. This implies that during the process of the decrease in temperature, the adsorption behavior of hydrogen molecules in the epoxy resin matrix becomes the dominant factor affecting its impermeability.

When the temperature further decreases to 20 K, the hydrogen permeation coefficients of the two epoxy resin systems decrease significantly. At this point, the influence of temperature on the hydrogen adsorption behavior is obviously weakened, and the diffusion coefficient shows an exponential decline. This abnormal phenomenon is likely to be closely related to the gas–liquid phase transition of hydrogen molecules. As the temperature approaches the critical liquefaction temperature of hydrogen, the high-energy adsorption sites (such as microporous structures) inside the epoxy resin will gradually become saturated, causing the growth rate of hydrogen adsorption to slow down significantly until adsorption equilibrium is reached [38,39]. Moreover, during the cooling process, high-energy adsorption sites (such as microvoids) will be preferentially occupied, and the remaining low-energy adsorption sites are less sensitive to temperature changes, which also leads to the adsorption effect becoming more stable with temperature changes.

The phase transition of hydrogen molecules significantly alters their physical properties: compared with gaseous molecules, the intermolecular forces of liquid molecules increase, and the viscosity and volume of the system also increase accordingly [40,41]. This phase transition enhances the hindrance effect on molecular motion, shortens the free motion path of hydrogen molecules, and consequently leads to an order-of-magnitude decrease in both the diffusion coefficient and the permeation coefficient.

#### 3.2.2. Effect of Curing Agents on Hydrogen Permeation

Compared with the DGEBA/DDM system, the DGEBA/PEA system exhibits significantly lower adsorption capacity and diffusion performance. The cause of this performance difference lies in the essential distinction between the molecular structures of the two curing agents: the PEA curing agent contains a relatively long flexible aliphatic chain segment, while the DDM curing agent has a rigid aromatic structure.

Analyzing from the perspective of adsorption characteristics, the rigid benzene ring structure in DDM can effectively maintain the spatial orientation of polar groups such as amino groups, thereby enhancing the interaction between these polar groups and hydrogen molecules, making the DDM curing system exhibit stronger adsorption capacity. In contrast, the flexible chain segment of PEA, due to its higher conformational freedom, may weaken the directional arrangement of polar groups, resulting in a decrease in the density of surface adsorption sites.

In terms of diffusion performance, although the flexible chain segment of PEA can enhance the mobility of local chain segments, its long-chain structure tends to form a complex entangled network of chain segments, which will significantly increase the tortuosity of the diffusion path. On the contrary, the rigid aromatic structure of DDM is conducive to forming a relatively regular three-dimensional network structure with larger molecular gaps, as shown in Figure 10. This structural feature provides more free volume and continuous channels for the diffusion of hydrogen molecules.

Based on the above research, it can be seen that the polymer network finally formed by the PEA curing system exhibits two key characteristics: one is the densified structure induced by the flexible chain; the other is the decrease in the concentration of surface polar groups. These factors work together, making the PEA curing system weaker than the DDM curing system in both the adsorption and diffusion characteristics of hydrogen permeation behavior, thus showing more excellent impermeability. This discovery provides an important theoretical basis for the subsequent regulation of the barrier performance of epoxy resins through molecular design.

### 3.3. Experimental Results

Constrained by the temperature characteristics of liquid nitrogen, the actual test temperature was precisely regulated and maintained at 100 K. At this specific temperature, the actual helium permeation amounts were meticulously recorded. For each material system, the three sets of measured data were subjected to a rigorous averaging process, yielding the mean permeation amounts for the DGEBA/PEA and DGEBA/DDM epoxy resin systems. Subsequently, these mean values were accurately transformed into gas permeation coefficients using the established formula, and the detailed results are presented in Table 3.

Based on the experimental data presented in Table 3, a striking disparity of 2–3 orders of magnitude is observed between the helium and hydrogen permeation coefficients for the DGEBA/PEA and DGEBA/DDM epoxy resin systems at a cryogenic temperature of 100 K. This significant divergence can be attributed to two primary factors.

Firstly, during the resin curing process, thermal cycling induces internal stresses that lead to micro-scale distortions within the molecular structure. Secondly, residual bubbles that remain unremoved during the pretreatment phase solidify into pore defects upon curing [42]. These structural imperfections act as preferential pathways for gas diffusion. Given that the atomic radius of helium (0.120 nm) is smaller than the molecular radius of hydrogen (0.128 nm), helium exhibits a greater propensity to diffuse through minuscule pores in low-temperature conditions.

Furthermore, the low temperature causes the material’s free volume to contract, which accentuates the selective permeation of small-molecule gases through the pores. This synergy of factors ultimately results in a substantial increase in the helium gas permeation coefficient, underscoring the critical role of molecular size and material microstructure in gas transport phenomena at cryogenic temperatures.

### 3.4. Effect of Material Defects on Hydrogen Permeation

To comprehensively elucidate the dynamic evolution mechanism of hydrogen molecule permeation behavior during the tensile failure of epoxy resin systems and validate the hypothesis proposed in the previous section—stating that microvoid defects in specimens enhance helium gas permeation—this study conducted uniaxial tensile failure simulations of epoxy resin systems. A specialized epoxy resin model, incorporating sample-preparation micro-defects, was constructed. Throughout the research, the dynamic response of the hydrogen molecule permeation coefficient during progressive tensile deformation was continuously monitored in real time. Particular emphasis was placed on analyzing the synergistic evolution patterns among micro-crack initiation, crack propagation, and the formation of hydrogen molecule permeation channels within the material. This investigation not only quantitatively uncovers the coupling mechanism between crack propagation and hydrogen molecule permeation performance but also provides crucial data to substantiate the theoretical conjecture regarding permeation enhancement induced by sample preparation defects.

**Figure 12:**
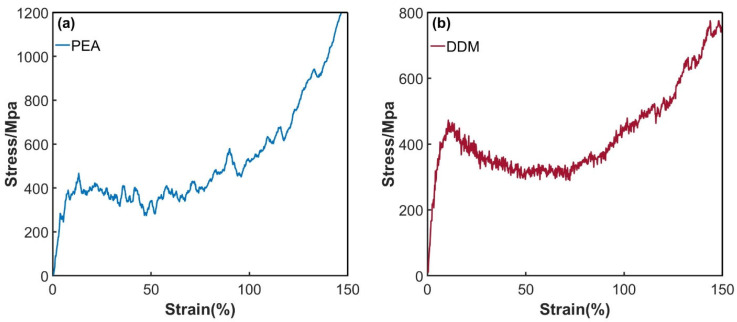
Tensile stress–strain curves of epoxy resin systems at a 100 K temperature: (**a**) PEA system; (**b**) DDM system.

During the simulation procedure, the Nose–Hoover temperature control method was initially employed to regulate the temperature of the hydrogen molecule permeation model to 100 K. Subsequently, tensile simulations were performed on the model box along the x, y, and z directions at a rate of $5\times 10^{8}/s$. By averaging the stress–strain responses across these three directions, the tensile stress–strain curves for the DGEBA/PEA and DGEBA/DDM epoxy resin systems at 100 K were derived, as depicted in Figure 11. Given that hydrogen molecule diffusion behavior predominantly governs the permeation performance of epoxy resins under isothermal conditions, this study standardized the solubility coefficient values. Specifically, the solubility coefficients of each system at 100 K and 0% tensile strain were utilized as the baseline for all subsequent calculations under varying strain conditions. Leveraging Equation (1), the study determined the trend of the permeation coefficients as a function of strain for the two epoxy resin systems during uniaxial tensile loading from 0% to 200% at 100 K. The results are illustrated in Figure 12, with detailed numerical data presented in Table 4 and Table 5.

**Figure 13:**
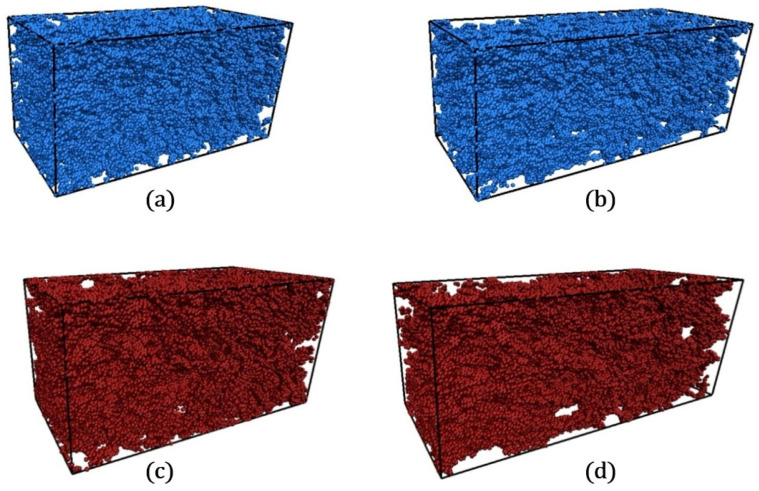
Atomic snapshots of epoxy resin system tensile, DGEBA/PEA system: (**a**) 50%; (**b**) 150%; DGEBA/DDM system: (**c**) 100%; (**d**) 150%.

The mechanical behavior at the crack tips of epoxy resin systems adheres to the generalized Hooke’s law. By analyzing the characteristics of the material’s stress–strain curves, the tensile process can be systematically divided into four distinct phases [43,44]. As illustrated in Figure 12 and Figure 13, when the strain is within the range of 0% to 50%, the system remains in the elastic yield stage. As the strain increases from 50% to 100%, it undergoes a transition from the yield state to the plastic flow stage. Between 100% and 200% strain, the system enters the plastic hardening phase. Once the strain exceeds 200%, the system ultimately succumbs to fracture failure.

**Figure 14:**
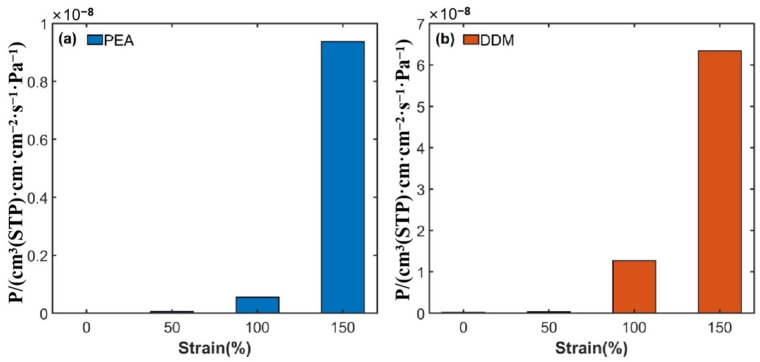
Variation in hydrogen molecule permeation coefficient with tensile strain at 100 K: (**a**) DGEBA/PEA system; (**b**) DGEBA/DDM system.

The evolution of the hydrogen molecule permeation coefficients with tensile strain for the two epoxy resin systems, as depicted in Figure 14, reveals a striking trend: when the strain surpasses 50%, the permeation coefficients increase by several orders of magnitude. This significant change can be attributed to the fact that upon entering the plastic flow stage, the slippage of molecular chains leads to a substantial rise in the proportion of irreversible strain, causing a sharp increase in the free volume fraction. These free volumes accumulate and coalesce over time, forming continuous diffusion channels. Although the topological reconstruction of the cross-linked network due to plastic flow generates new energy barriers, and the dynamic equilibrium between stress hardening and softening increases the tortuosity of the diffusion pathways, partially offsetting the enhanced permeation effect, the impact of pathway tortuosity diminishes as the strain continues to escalate, resulting in an accelerated growth rate of the permeation coefficients.

**Figure 15:**
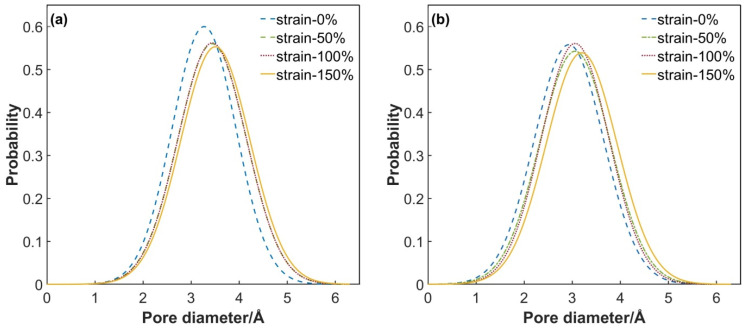
Evolution curve of PSD of system with tensile strain: (**a**) PEA system; (**b**) DDM system.

Micro-cracks initiate within the epoxy resin systems when the strain reaches 80%. As the strain further intensifies, the chemical bonds within the cross-linked network break, giving rise to numerous nanoscale pores. Through continuous merging and expansion, these pores not only extend and widen the continuous permeation channels but also reduce the tortuosity of these channels, significantly enhancing the diffusion efficiency of hydrogen molecules. A comparison between the experimental data in Table 3 and the results of molecular dynamics simulations demonstrates excellent agreement when the tensile strain reaches 100%. This congruence can be elucidated by the microscopic mechanisms revealed in the PSD distribution (Figure 15). At this strain level, the probability density of pore sizes within the material peaks at 3–3.5 Å, with the maximum pore size remaining below 6 Å. The dynamic coalescence of these nanoscale pores not only substantially enlarges the free volume but also constructs an efficient diffusion network, providing unobstructed pathways for hydrogen molecule permeation. Once these pores merge into macroscopic cracks, the system enters the unstable failure stage, with the permeation coefficients increasing exponentially and exceeding the safety threshold, ultimately leading to the complete breakdown of the material’s sealing performance.

The foregoing analysis clearly demonstrates that, in the construction of a molecular dynamics simulation model for the hydrogen molecule permeation behavior of epoxy resins, a systematic consideration of the influence mechanism of material processing defects on permeation performance is indispensable. These defects predominantly originate from thermal cycling effects and the evolution of microbubbles during the curing process, and their propagation is intricately intertwined with the plastic deformation stage of the material.

Consequently, it is strongly recommended to integrate the dynamic evolution laws of plastic flow within the strain range of 100–150% into the model-building process. This specific strain interval marks the crucial transition phase of the material from plastic hardening to fracture failure. During this stage, the progressive accumulation of molecular chain fractures is particularly pronounced, triggering a rapid increase in free volume and a significant reconstruction of the tortuosity of the diffusion channel network.

Through the replication of the coupled process of molecular chain fractures and free volume evolution within this strain range in numerical simulations, it becomes feasible to effectively mimic the microstructural alterations induced by processing defects in actual materials, encompassing the formation, expansion, and interlinking of pores. This modeling methodology, which is founded on the coupled mechanism of plastic deformation and defect evolution, not only enables an accurate reproduction of the impact of processing defects on hydrogen molecule permeation behavior but also offers pivotal theoretical underpinnings for the establishment of cross-scale correlation models between experimental observations and simulation predictions.

## 4. Conclusions

This study conducted a comprehensive and systematic investigation into the mechanisms by which the cross-linking degree, permeation environment temperature, and molecular structure of curing agents influence the hydrogen permeation behavior of epoxy resin systems. The research findings indicate a pronounced positive correlation between the three-dimensional network cross-linking degree of the resin systems and their hydrogen barrier capabilities. As the cross-linking degree increases, the free volume fraction within the systems decreases substantially. This reduction effectively restricts the connectivity of hydrogen molecule diffusion pathways, thereby significantly enhancing the materials’ resistance to hydrogen permeation.

Temperature exerts a profound influence on hydrogen permeation behavior. In low-temperature environments, epoxy resin systems exhibit an enhanced capacity to adsorb hydrogen molecules. While this increased adsorption promotes the migration of hydrogen molecules within the material, potentially exacerbating permeation, a notable nonlinear change occurs in the permeation coefficient when the temperature reaches the phase transition temperature of hydrogen molecules.

A comparative analysis of the effects of different curing agent molecular structures reveals that, compared to the DDM curing agent with its rigid structure, the flexible chain segments of the PEA curing agent can reduce the hydrogen permeation coefficient by one to two orders of magnitude. The flexible chain segments enhance the mobility of molecular chains, enabling the polymer network to form a more densely packed structure. This densification reduces the free volume and constricts diffusion channels, thereby markedly improving the materials’ hydrogen barrier performance.

Furthermore, this study leveraged molecular dynamics simulations to develop a micro-scale model that incorporates manufacturing defects. By introducing a dynamic strain evolution algorithm, the model simulated the deformation processes of materials under real-world operating conditions. This approach effectively minimized the discrepancies between simulated and experimental values of the permeation coefficient, providing a robust theoretical framework and practical techniques for accurately predicting the hydrogen permeation performance of epoxy resins in complex environments.

## Figures and Tables

**Table 1:** Simulated values of D, S, and P for liquid hydrogen molecule in PEA epoxy resins system with different degrees of cross-linking.

Degree of Crosslinking /%	D $/{cm}^{2}\cdot s^{-1}$	S $/{cm}^{3}\;(STP)\cdot {cm}^{-3}\cdot {Pa}^{-1}$	P $/{cm}^{3}\;(STP)\cdot cm\cdot {cm}^{-2}\cdot s^{-1}\cdot {Pa}^{-1}$
70	$1.433{\;\times \;10}^{-6}$	$9.042{\;\times \;10}^{-7}$	${1.293\;\times \;10}^{-12}$
75	$9.467{\;\times \;10}^{-7}$	$1.247{\;\times \;10}^{-6}$	${1.184\;\times \;10}^{-12}$
80	$8.900{\;\times \;10}^{-7}$	$1.322{\;\times \;10}^{-6}$	${1.175\;\times \;10}^{-12}$
85	$3.700{\;\times \;10}^{-7}$	$1.430{\;\times \;10}^{-6}$	${5.291\;\times \;10}^{-13}$
90	$2.229{\;\times \;10}^{-7}$	$1.560{\;\times \;10}^{-6}$	${3.479\;\times \;10}^{-13}$

**Table 2:** Simulated values of D, S, and P for liquid hydrogen molecule in DDM epoxy resins system with different degrees of cross-linking.

Degree of Crosslinking /%	D $/{cm}^{2}\cdot s^{-1}$	S $/{cm}^{3}(STP)\cdot {cm}^{-3}\cdot {Pa}^{-1}$	P $/{cm}^{3}(STP)\cdot cm\cdot {cm}^{-2}\cdot s^{-1}\cdot {Pa}^{-1}$
70	$2.736{\;\times \;10}^{-5}$	$1.231{\;\times \;10}^{-6}$	${3.370\;\times \;10}^{-11}$
75	$1.463{\;\times \;10}^{-5}$	$1.385{\;\times \;10}^{-6}$	${2.015\;\times \;10}^{-11}$
80	$1.217{\;\times \;10}^{-5}$	$1.519{\;\times \;10}^{-6}$	${1.854\;\times \;10}^{-11}$
85	$1.050{\;\times \;10}^{-5}$	$1.687{\;\times \;10}^{-6}$	${1.775\;\times \;10}^{-11}$
90	$8.500{\;\times \;10}^{-6}$	$1.715{\;\times \;10}^{-6}$	${1.454\;\times \;10}^{-11}$

**Table 3:** Helium leakage test value of PEA and DDM epoxy resin system.

Sample	L $/{Pa\cdot m}^{3}\cdot s^{-1}$	$L_{average}$ $/{Pa\cdot m}^{3}\cdot s^{-1}$	P $/{cm}^{3}(STP)\cdot cm\cdot {cm}^{-2}\cdot s^{-1}\cdot {Pa}^{-1}$
A-1	$1.50{\;\times \;10}^{-8}$		
A-2	$1.49{\;\times \;10}^{-8}$	$1.52{\;\times \;10}^{-8}$	$2.56{\;\times \;10}^{-9}$
A-3	$1.56{\;\times \;10}^{-8}$		
B-1	$1.85{\;\times \;10}^{-8}$		
B-2	$1.76{\;\times \;10}^{-8}$	$1.90{\;\times \;10}^{-8}$	$3.20{\;\times \;10}^{-9}$
B-3	$2.09{\;\times \;10}^{-8}$		

**Table 4:** Simulated values of permeation coefficients of PEA epoxy resin systems under different tensile strains at 100 K.

System	Strain /%	P $/{cm}^{3}(STP)\cdot cm\cdot {cm}^{-2}\cdot s^{-1}\cdot {Pa}^{-1}$
	0	$3.74{\;\times \;10}^{-12}$
	50	$5.66{\;\times \;10}^{-11}$
PEA system	100	$5.56{\;\times \;10}^{-10}$
	150	$9.38{\;\times \;10}^{-9}$
	200	$1.55{\;\times \;10}^{-8}$

**Table 5:** Simulated values of permeation coefficients of DDM epoxy resin systems under different tensile strains at 100 K.

System	Strain /%	P $/{cm}^{3}(STP)\cdot cm\cdot {cm}^{-2}\cdot s^{-1}\cdot {Pa}^{-1}$
	0	$2.37{\;\times \;10}^{-10}$
	50	$3.24{\;\times \;10}^{-10}$
DDM system	100	$1.27{\;\times \;10}^{-8}$
	150	$6.34{\;\times \;10}^{-8}$
	200	$7.02{\;\times \;10}^{-8}$

## Data Availability

The data presented in this study are available on request from the corresponding author.
